# Outcomes of Human Metapneumovirus Infections in Nursing Home Residents: A Matched Cohort Analysis

**DOI:** 10.1093/infdis/jiaf151

**Published:** 2025-07-16

**Authors:** Nidhi Bhaskar, Yasin Abul, Frank DeVone, Kevin W McConeghy, Tayler Leonard, Christopher W Halladay, Stefan Gravenstein, James L Rudolph

**Affiliations:** Center of Innovation in Long Term Services and Supports, Providence VA Medical Center, Providence, Rhode Island, USA; Division of Geriatric and Palliative Medicine, Warren Alpert Medical School of Brown University, Providence, Rhode Island, USA; Warren Alpert Medical School of Brown University, Providence, Rhode Island, USA; Center of Innovation in Long Term Services and Supports, Providence VA Medical Center, Providence, Rhode Island, USA; Division of Geriatric and Palliative Medicine, Warren Alpert Medical School of Brown University, Providence, Rhode Island, USA; Center of Innovation in Long Term Services and Supports, Providence VA Medical Center, Providence, Rhode Island, USA; Center of Innovation in Long Term Services and Supports, Providence VA Medical Center, Providence, Rhode Island, USA; Department of Health Services, Policy and Practice, School of Public Health, Brown University, and Providence, Rhode Island, USA; North Carolina Agricultural and Technical State University, Greensboro, North Carolina, USA; Center of Innovation in Long Term Services and Supports, Providence VA Medical Center, Providence, Rhode Island, USA; Center of Innovation in Long Term Services and Supports, Providence VA Medical Center, Providence, Rhode Island, USA; Division of Geriatric and Palliative Medicine, Warren Alpert Medical School of Brown University, Providence, Rhode Island, USA; Department of Health Services, Policy and Practice, School of Public Health, Brown University, and Providence, Rhode Island, USA; Center of Innovation in Long Term Services and Supports, Providence VA Medical Center, Providence, Rhode Island, USA; Division of Geriatric and Palliative Medicine, Warren Alpert Medical School of Brown University, Providence, Rhode Island, USA; Department of Health Services, Policy and Practice, School of Public Health, Brown University, and Providence, Rhode Island, USA

**Keywords:** human metapneumovirus, influenza, aged, nursing home, respiratory infection

## Abstract

**Background:**

Identified in 2001, human metapneumovirus (hMPV) infection can lead to hospitalization and death, especially in older adults and children.

**Methods:**

This retrospective, propensity-matched study compared cases of hMPV infection with cases of influenza virus and respiratory syncytial virus (RSV) infection for hospitalization and death outcomes in older adults living in Community Living Centers (CLCs), nursing homes operated by the US Department of Veterans Affairs. We evaluated electronic medical records from July 2017 to June 2024. We included CLC residents with laboratory-confirmed hMPV, influenza virus, or RSV infections. The propensity-matched analysis balanced demographic and clinical characteristics. Proportional hazards models estimated the risk of hospitalization, death or the combined outcome over the 90 days after diagnosis.

**Results:**

We identified 178 hMPV, 1379 influenza virus, and 681 RSV laboratory-confirmed infections. In the propensity analysis. Residents with hMPV infection (n = 173) were well matched to those with influenza virus infection (n = 746). The matched cohort proportional hazard analysis (hMPV vs influenza virus infection) showed similar hazards for 90-day outcomes of hospitalization (adjusted hazard ratio, 1.00 [95% confidence interval, .67–1.49]), death (0.79 [.48–1.29]), or both combined (0.81 [.58–1.12]). With use of similar methods, residents with hMPV (n = 170) were well matched to those with RSV (n = 437). The matched cohort proportional hazard analysis showed similar 90-day outcomes of hospitalization (adjusted hazard ratio, 1.00 [95% confidence interval, .66–1.51)], death (0.93 [.58–1.56], or both combined (0.97 [.68–1.37]).

**Conclusions:**

Resident infection with hMPV produced similar likelihoods of hospitalization and death as infection with influenza virus or RSV. Increased understanding of hMPV and appropriate testing are needed to accurately detect, prevent, and manage hMPV in nursing homes.

Nursing home (NH) residents have substantial morbidity and mortality risks associated with respiratory virus infections. The 4 most prevalent respiratory viruses that infect older adults are influenza virus, seasonal coronaviruses, respiratory syncytial virus (RSV), and human metapneumovirus (hMPV) [1–4]. RSV and hMPV may seem “less threatening” because of the high frequency of self-limited, mild clinical disease in adolescents and younger adults [5]. However, these viruses pose significant health threats to young children, older adults, and, particularly, those with underlying conditions, such as chronic obstructive pulmonary disease (COPD), asthma, and cancer [6]. As with influenza virus and RSV infections, hMPV infections in young children and older adults may result in higher risks of hospitalization and death [7].

Isolated in 2001 from children in the Netherlands, hMPV has particular importance for NH residents. First, NH residents have personal care needs (eg, dressing, grooming, and bathing) requiring close support from healthcare personnel, and transmission of hMPV occurs by direct or close contact with contaminated secretions, including droplets or fomites [8]. In addition, NH living is communal, with a focus on social engagement, shared meals, and joint recreational activities, which allow viral spread. Next, NH residents are particularly vulnerable to stressors from comorbid conditions and functional and cognitive deficits. hMPV causes bronchiolitis and pneumonia, which, in a highly vulnerable population, can lead to hospitalizations, neurological symptoms, and death [9]. The clinical presentation of hMPV infection mimics that of other respiratory viruses, and symptoms in NH residents vary widely, from asymptomatic cases to severe life-threatening conditions [10]. Increased use of respiratory panel-based, multiplex diagnostic assays has enhanced hMPV identification. However, NHs generally lack basic laboratory facilities and laboratory-trained personnel required for diagnostic testing.

Because of the increasing awareness of hMPV in older patients and the vulnerability of NH residents, we undertook a cohort study of NH residents in an integrated health system, the US Department of Veterans Affairs (VA) Community Living Centers (CLCs). The VA owns and operates the 134 CLCs and provides comprehensive care to veterans with skilled nursing needs. The VA CLCs have an average daily census of nearly 6000, and they serve >24 000 unique veterans annually [11]. We hypothesized that residents infected with hMPV would have clinical characteristics different from those seen with influenza virus infection and that, with propensity matching, they experience hospitalization and death comparably to those infected with influenza virus and RSV. Secondarily, we compared the seasonal patterns of hMPV, RSV, and influenza virus infections.

## METHODS

### Ethics

This secondary analysis was approved by the Providence VA Medical Center’s institutional review board and informed consent was waived. In accordance with privacy rules, variables and outcomes with <10 residents are not reported.

### Cohort

Using VA electronic medical records, we identified all residents of VA CLCs from 1 July 2017 to 30 June 2024. We included all residents recorded as spending ≥1 night in one of the 134 VA CLCs. There are only 3 states without CLCs (Rhode Island, Vermont, and Hawaii). Past work identified that CLC residents have a high rate (>95%) of vaccination against influenza [12].

### Virus Identification

VA laboratory records were used to identify all laboratory tests that detected hMPV, influenza A or B, or RSV during the period of interest. The VA electronic medical record does not discriminate between a panel test or single virus test. For this study, those included were residents of the CLC at the time of testing. To our knowledge, there was not a consistent protocol for respiratory virus testing across CLCs during the years of this study, and we are unable to comment whether testing was performed for symptoms or outbreak management. We used the same-day reporting of diagnostic results (positive or negative) for respiratory viruses (influenza virus, RSV, and hMPV) as evidence of a panel test. All VA laboratories are Clinical Laboratory Improvement Amendments (CLIA) certified and Joint Commission accredited. From the electronic medical record, we were unable to detail test-specific equipment or manufacturers.

### Outcomes

The primary outcomes of interest included hospitalization and death due to any cause in the 90 days after the date of the positive diagnostic test. Hospitalization was recorded as a transfer from the CLC to an inpatient bed section. The proximity of CLCs to hospitals allows transfer of patients requiring hospitalization on the same day or next day. To identify non-VA hospitalization transfer from CLCs, we used VA payments sources to identify occurrences of “VA-paid community hospitalization.” Death was identified with the VA source file, a compilation of multiple administrative sources of data, including the electronic health record, VA benefits records, and social security records. Outcomes were truncated at 90 days. We selected 90 days of follow-up to identify secondary complications of respiratory infections, common among frail older NH populations that lead to hospitalization or death (eg, pneumonia or cardiac event).

### Covariates

Demographic data were collected from VA administrative databases. Race, a social construct, was selected by the resident on VA enrollment. *International Classification of Diseases, Tenth Revision, Clinical Modification* (*ICD-10-CM*) codes from inpatient and outpatient VA encounters were used to calculate the Elixhauser Comorbidity Index (ECI) in the year before testing [13]. The Agency for Healthcare Research and Quality has coordinated the public-private adaptation of the current ECI, including the 38 categories of *ICD-10-CM* diagnoses and the validation for in-hospital mortality, 90-day mortality, and 90-day readmission [14]. Dementia is not explicitly included in the ECI. However, because dementia is prevalent in the NH population, we added dementia diagnosis *ICD-10-CM* codes to the list of collected comorbid conditions [15]. COPD was separated from the “pulmonary” category of the ECI for display purposes, given the high prevalence of COPD in the veteran population. Analytically, however, we retained the pulmonary category of the ECI.

### Analysis

The date and result of viral testing for hMPV, influenza virus, and RSV were recorded during the CLC stay with the proportion of monthly positive test results reported annually and cumulatively.

#### Propensity Matching

Propensity matching is an analytic method that reduces bias by balancing observed differences in comparison populations [16]. This study used radius propensity matching, which selects all matches (influenza virus or RSV infection cases) within a specified radius (caliper width, 0.30) of the hMPV propensity score. Radius matching allows for multiple matches per hMPV while avoiding poor matches, which improves the balance between comparison groups.

We performed separate propensity-matched comparisons of hMPV with influenza virus and hMPV with RSV. The propensity matching used demographic and ECI variables to create a propensity score for each resident with influenza virus and separately with RSV. For each hMPV case, up to 4 cases of influenza virus or RSV infection within a propensity score caliper of 0.30 were selected. A caliper is a statistical ring around a case to identify potential matches. Narrower calipers reduce both matched cases. Cases of influenza virus or RSV infection outside the caliper are discarded for the hMPV case. Infection year was forced into the matching to account for seasonal changes in immunity and virulence (eg, 2022–2023 hMPV cases were matched with 2022–2023 influenza or RSV cases) and the changes in CLC population during the coronavirus disease 2019 (COVID-19) pandemic [17]. Before and after propensity matching, variables from CLC residents with positive hMPV and positive influenza virus/RSV test results were compared using the standardized mean differences (SMDs). This method was chosen because it provides a measure of effect size standardized by the standard deviation (SD) of the variable. SMD values <0.2 represent a small effect size and are used as a rule of thumb for comparing matched cohorts [18, 19]. Descriptive tables present percentages with numbers for dichotomous variables and means with SDs for continuous variables.

#### Proportional Hazards Modeling

Kaplan-Meier curves of the matched cohorts were produced for the 90-day outcomes of hospitalization, death, and both combined. Subsequently, we performed proportional hazards modeling for a I estimate (unmatched) of the relative hazard of hospitalization/death and a propensity-matched set (matched) for the hazard ratio (HR) of hMPV infection relative to the influenza or RSV comparison group. An HR >1.0 suggests a more common outcome among residents with hMPV. We present all HRs with their 95% confidence intervals (CIs).

## RESULTS

**Figure 1.**
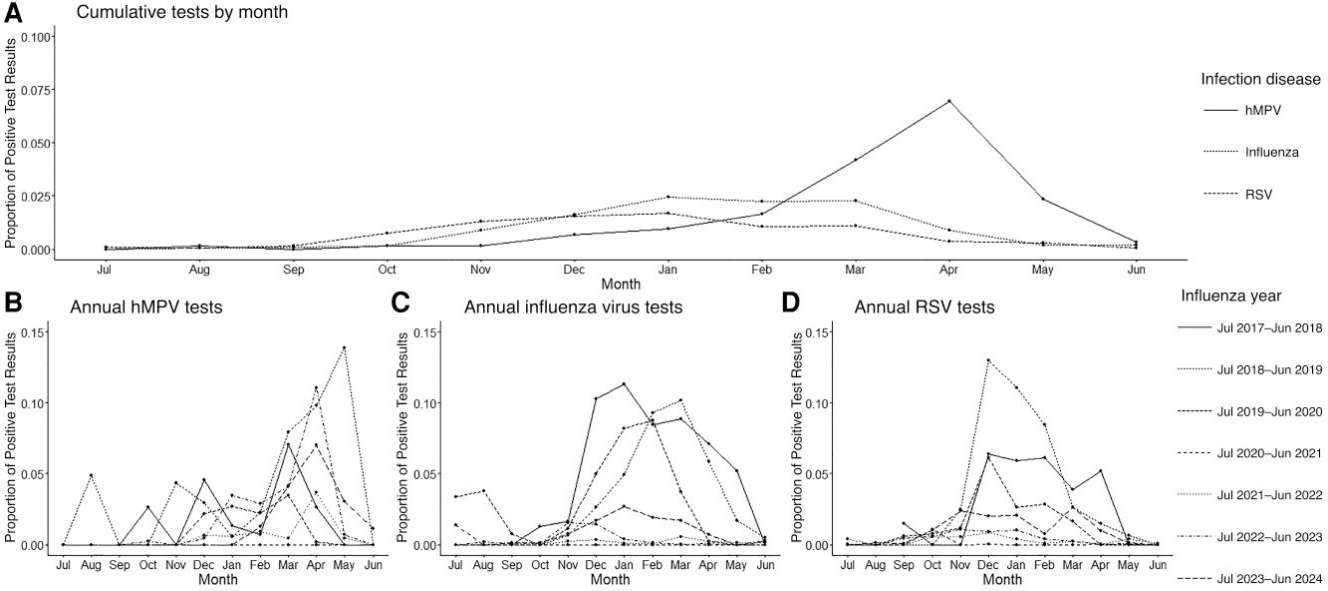
Positive human metapneumovirus (hMPV), influenza virus, and respiratory syncytial virus (RSV) test results by month. For hMPV and influenza virus, we examined the proportion of positive tests relative to the total number of tests done in that month. *A,* hMPV data over all years of the study (except the year of coronavirus disease 2019 [COVID-19], July 2020 to June 2021) *B–D*, Data for each year individually for hMPV (*B*), influenza virus (*C*) and RSV) (*D*). The infection control practices of the COVID-19 year resulted in a markedly low number of positive tests for all viruses and were thus excluded from the cumulative analysis in *A*.

Figure 1 describes the cumulative proportion (Figure 1*A*) of positive test results by months for hMPV, influenza virus, and RSV. The year of July 2020 to July 2021 (during the COVID-19 pandemic) is excluded from the cumulative proportion (Figure 1*A*) due to low positivity for the other viruses. The other panels present the annual breakout of positive results for hMPV (Figure 1*B*), influenza virus (Figure 1*C*), and RSV (Figure 1*D*). The hMPV seasonal peak appears after the seasonal influenza peak. Influenza and RSV appear seasonally similar.

### hMPV and Influenza Virus

Over the study period, 19 302 hMPV tests and 110 125 influenza virus tests were performed. In 173 of 178 residents, positive hMPV test results occurred on the same day as influenza virus tests, suggesting that hMPV testing resulted from a panel. No coinfections were identified, suggesting the specificity of the test to each virus.

Table 1 describes the unmatched population of the CLC residents with hMPV (n = 178) and influenza virus (n = 1379) detection, as well as the propensity-matched cohorts for hMPV (n = 170) and influenza (n = 746). In general, both populations were older (mean age [SD], 76.3 [10.2] years for hMPV vs 74.3 [11.0] years for influenza virus; SMD, 0.18) and predominantly male (96.6% vs 96.5% male, respectively; SMD, 0.01). The comorbid conditions were generally matched at baseline, with more COPD (31.4% for hMPV vs 20.9% for influenza; SMD, 0.24) and less renal disease (16.8% vs 27.0%, respectively; SMD, 0.25) in those with hMPV. The comorbid conditions demonstrated a balanced ECI burden (mean ECI [SD], 6.1 [3.7] for hMPV vs 6.3 [3.4] for influenza; SMD, 0.05)

**Table 1.** Characteristics of Community Living Center Residents With Human Metapneumovirus or Influenza Virus

Variable	CLC Residents, No. (%)^a^					
	Before Matching			After Matching		
	hMPV (n = 178)	Influenza Virus (n = 1379)	SMD	hMPV (n = 173)	Influenza Virus (n = 746)	SMD
Age, mean (SD), y	76.26 (10.17)	74.31 (10.98)	0.18	75.96 (10.00)	74.86 (10.52)	0.1074
Male sex	172 (96.63)	1331 (96.52)	0.006	167 (96.53)	721 (96.65)	0.0064
White race	129 (72.47)	1014 (73.53)	0.024	125 (72.25)	542 (72.65)	0.0089
Married status	73 (41.01)	528 (38.29)	0.06	70 (40.46)	283 (37.94)	0.0518
DM	90 (50.56)	685 (49.67)	0.01	86 (49.71)	374 (50.13)	0.0085
Hypertension	123 (69.10)	1,043 (75.63)	0.14	121 (69.94)	552 (73.99)	0.0903
CHF	49 (27.53)	419 (30.38)	0.06	48 (27.75)	215 (28.82)	0.0239
Pulmonary disease	76 (42.70)	546 (39.59)	0.06	73 (42.20)	325 (43.57)	0.0277
COPD	56 (31.46)	288 (20.88)	0.24	54 (31.21)	218 (29.22)	0.0434
Valvular disease	22 (12.36)	158 (11.46)	0.02	20 (11.56)	73 (9.79)	0.0575
Alcohol use disorder	20 (11.24)	150 (10.88)	0.01	19 (10.98)	86 (11.53)	0.0173
Substance use disorder	17 (9.55)	111 (8.05)	0.05	17 (9.83)	70 (9.38)	0.0150
Anemia	73 (41.01)	608 (44.09)	0.06	70 (40.46)	323 (43.30)	0.0575
Depression	73 (41.01)	555 (40.25)	0.01	70 (40.46)	305 (40.88)	0.0086
Tumor	35 (19.66)	222 (16.10)	0.09	32 (18.50)	118 (15.82)	0.0711
Psychoses	48 (26.97)	356 (25.82)	0.02	46 (26.59)	206 (27.61)	0.0230
Renal disease	30 (16.85)	373 (27.05)	0.25	30 (17.34)	136 (18.23)	0.0233
Dementia	97 (54.49)	745 (54.02)	0.009	94 (54.34)	412 (55.23)	0.0179
AF	58 (32.58)	392 (28.43)	0.09	55 (31.79)	221 (29.62)	0.0470
TBI	12 (6.74)	80 (5.80)	0.03	11 (6.36)	48 (6.43)	0.0031
ECI, mean (SD)	6.13 (3.71)	6.32 (3.37)	0.05	6.05 (3.66)	6.16 (3.34)	0.0312

Abbreviations: AF, atrial fibrillation; CHF, congestive heart failure; CLC, Community Living Center; COPD, chronic obstructive pulmonary disease; DM, diabetes mellitus; ECI, Elixhauser Comorbidity Index; hMPV, human metapneumovirus; SD, standard deviation; SMD, standardized mean difference; TBI, traumatic brain injury.

^a^Data represent no (%) of CLC residents unless otherwise specified.

The propensity algorithm matched 170 residents with hMPV to 746 with influenza. Eight hMPV and 633 influenza cases did not match. There was balance in age (mean age [SD], 76.0 [10.0] years for hMPV vs 74.9 [10.5] years for influenza; SMD, 0.11), with similar ECIs (mean [SD], 6.0 [3.7] vs 6.2 [3.3], respectively; SMD, 0.03), as well as similar prevalences of pulmonary disease, COPD, renal disease, dementia, and substance use disorder. Importantly, the propensity-matched cohorts for hMPV and influenza are well balanced, with all SMDs for demographics and comorbid conditions ≤0.1.

### hMPV and RSV

Over the course of the study, 19 302 hMPV and 98 690 RSV tests were performed. Table 2 describes the unmatched population of those residents with hMPV (n = 178) and RSV (n = 681), as well as the propensity-matched cohorts for hMPV (n = 170) and RSV (n = 437). Both hMPV- and RSV-infected populations were older (mean age [SD], 76.3 [10.2] years for hMPV vs 76.8 [10.5] years for RSV; SMD, 0.0505) and predominantly male (96.63% vs 96.18%, respectively; SMD, 0.024). The comorbid conditions were generally balanced at baseline, with notable exceptions, such as increased COPD in those with hMPV (31.46% for hMPV vs 23.64% for RSV; SMD, 0.1757). The comorbid conditions also demonstrated a balanced ECI burden (mean ECI [SD], 6.13 [3.71] for hMPV vs 6.38 [3.47] for RSV; SMD, 0.0688).

**Table 2.** Characteristics of Community Living Center Residents With Human Metapneumovirus and Respiratory Syncytial Virus

Variable	CLC Residents, No. (%)^a^					
	Before Matching			After Matching		
	hMPV (n = 178)	RSV (n = 681)	SMD	hMPV (n = 170)	RSV (n = 437)	SMD
Age, mean (SD), y	76.26 (10.17)	76.78 (10.55)	0.0505	76.44 (9.80)	76.86 (10.40)	0.0420
Male sex	172 (96.63)	655 (96.18)	0.024	164 (96.47)	425 (97.25)	0.0449
White race	129 (72.47)	507 (74.45)	0.0448	125 (73.53)	330 (75.51)	0.0456
Married status	73 (41.01)	244 (35.83)	0.1067	68 (40.00)	157 (35.93)	0.0840
DM	90 (50.56)	348 (51.10)	0.0108	89 (52.35)	217 (49.66)	0.0540
Hypertension	123 (69.10)	504 (74.01)	0.1089	120 (70.59)	300 (68.65)	0.0422
CHF	49 (27.53)	215 (31.57)	0.0887	48 (28.24)	119 (27.23)	0.0224
Pulmonary disease	76 (42.70)	245 (35.98)	0.1379	70 (41.18)	166 (37.99)	0.0653
COPD	56 (31.46)	161 (23.64)	0.1757	52 (30.59)	120 (27.46)	0.0690
Valvular disease	22 (12.36)	83 (12.19)	0.0052	21 (12.35)	44 (10.07)	0.0724
Alcohol use disorder	20 (11.24)	66 (9.69)	0.0505	19 (11.18)	42 (9.61)	0.0513
Substance use disorder	17 (9.55)	63 (9.25)	0.0103	17 (10.00)	39 (8.92)	0.0368
Anemia	73 (41.01)	311 (45.67)	0.0941	72 (42.35)	186 (42.56)	0.0042
Depression	73 (41.01)	273 (40.09)	0.0188	71 (41.76)	173 (39.59)	0.0443
Tumor	35 (19.66)	112 (16.45)	0.0837	34 (20.00)	71 (16.25)	0.0975
Psychoses	48 (26.97)	197 (28.93)	0.0437	47 (27.65)	126 (28.83)	0.0263
Renal disease	30 (16.85)	162 (23.79)	0.173	29 (17.06)	75 (17.16)	0.0028
Dementia	97 (54.49)	418 (61.38)	0.1398	94 (55.29)	250 (57.21)	0.0386
AF	58 (32.58)	206 (30.25)	0.0503	55 (32.35)	133 (30.43)	0.0413
TBI	12 (6.74)	38 (5.58)	0.0483	11 (6.47)	20 (4.58)	0.0830
ECI, mean (SD)	6.13 (3.71)	6.38 (3.47)	0.0688	6.25 (3.70)	5.92 (3.40)	0.0921

Abbreviations: AF, atrial fibrillation; CHF, congestive heart failure; CLC, Community Living Center; COPD, chronic obstructive pulmonary disease; DM, diabetes mellitus; ECI, Elixhauser Comorbidity Index; hMPV, human metapneumovirus; RSV, respiratory syncytial virus; SD, standard deviation; SMD, standardized mean difference; TBI, traumatic brain injury.

^a^Data represent no (%) of CLC residents unless otherwise specified.

In the propensity-matched cohorts, 170 residents with hMPV were matched to 437 residents with RSV. Eight residents with hMPV and 244 with RSV did not match. The matching process provided a well-balanced comparison, with all SMD values for demographics and comorbid conditions ≤0.1. After matching, the age (mean [SD], 76.44 [9.80] years for hMPV vs 76.86 [10.40] years for RSV; SMD, 0.042) and ECI (6.25 [3.70] vs 5.92 [3.40], respectively; SMD, 0.0921) remained well balanced between groups, along with similar prevalence rates of pulmonary disease, dementia, and substance use disorders (Table 2).

### Hospitalization and Mortality Outcomes

**Figure 2.**
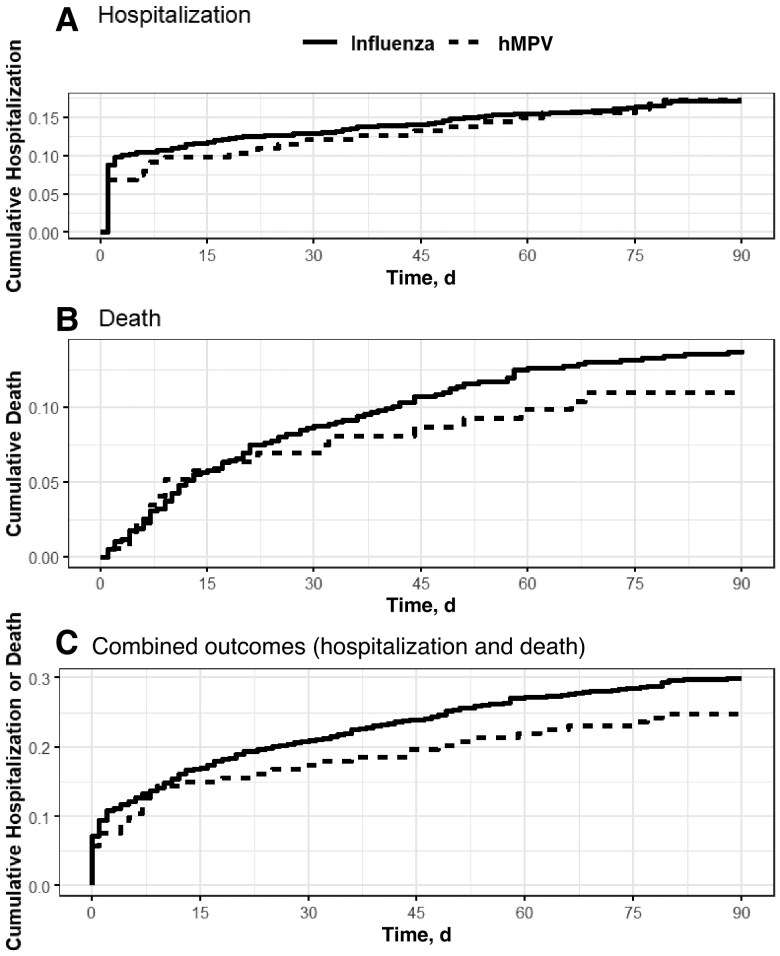
Time-to-outcome (Kaplan-Meier) curves for human metapneumovirus (hMPV) and influenza virus infection, for hospitalization (*A*), death (*B*), and combined outcomes (*C*). The a priori event time was 90 days.

The hMPV-influenza propensity-matched cohort shows similar risks for hospitalization (17.3% [n = 30] for hMPV vs 17.1% [n = 128] for influenza), death (10.9% [n = 19] vs 13.8% [n = 103]), and the combined outcome (24.9% [n = 43] vs 29.9% [n = 223]) (Table 3). Proportional hazard analysis on the hMPV-influenza propensity-matched cohort found similar hazard rates for hospitalization (adjusted HR, 1.00 [95% CI, .67–1.49]), death (0.79 [.48–1.29]), and the combined outcome (0.81 [.58–1.12]). Figure 2 presents the associated Kaplan-Meier curves for hospitalization (Figure 2*A*), death (Figure 2*B*), and both outcomes (Figure 2*C*).

**Table 3.** 90-Day Outcomes of Human Metapneumovirus Relative to Influenza Virus Infection

Outcome	No. with Outcome (matched cohort)		HR (95% CI)^a^	
	hMPV Infection (n = 173)	Influenza Virus Infection (Reference) (n = 746)	Unmatched	Matched
Hospitalization	30	128	1.10 (.76–1.61)	1.00 (.67–1.49)
Death	19	103	.88 (.55–1.40)	.79 (.48–1.29)
Hospitalization and death combined	43	223	.93 (.68–1.26)	.81 (.58–1.12)

Abbreviations: CI, confidence interval; hMPV, human metapneumovirus; HR, hazard ratio.

^a^The HR represents the hazard of the hMPV cohort relative to the influenza virus cohort.

**Figure 3.**
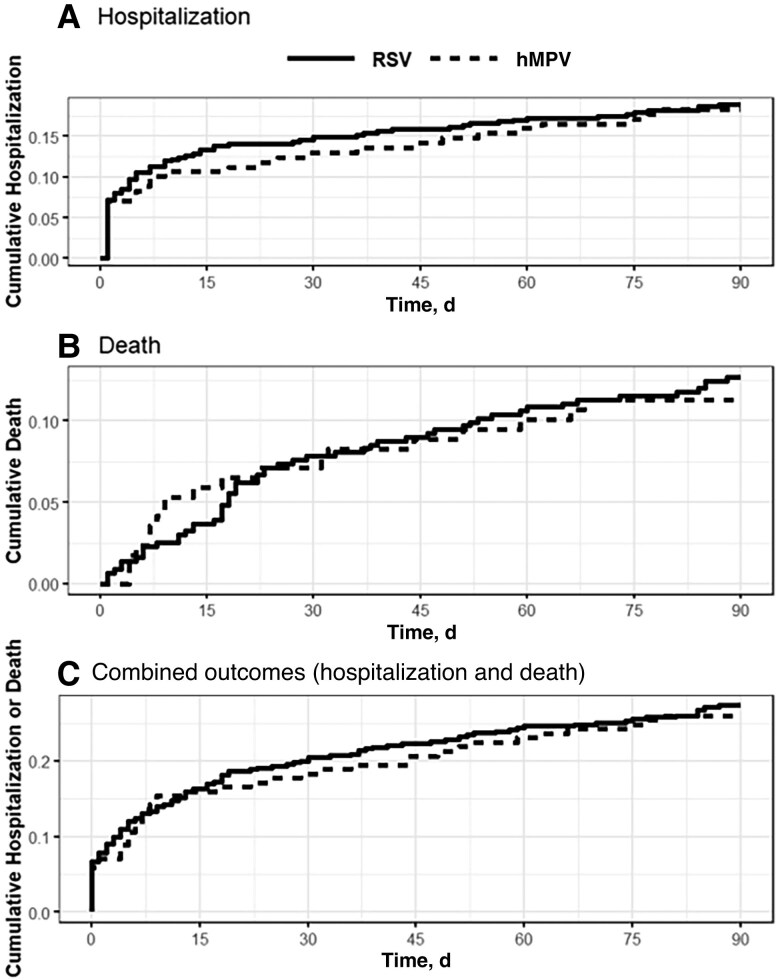
Time-to-outcome (Kaplan-Meier) curves for human metapneumovirus (hMPV) and respiratory syncytial virus (RSV) infection, for hospitalization (*A*), death (*B*), and combined outcomes (*C*). The a priori event time was 90 days.

Comparing hMPV and RSV, the matched cohorts demonstrate similar risks for hospitalization (18.2% [n = 31] for hMPV vs 18.0% [n = 79] for RSV), death (11.1% [n = 19] vs 12.1% [n = 53]), and the combined outcome (25.9% [n = 44] vs 26.5% [n = 116]) (Table 4). The hMPV-RSV proportional hazard analysis identified similar adjusted hazard for hospitalization (adjusted HR, 1.00 [95% CI, .66–1.51]), death (0.93 [.55–1.56]), and the combined outcome (0.97 [.68–1.37]). Figure 3 displays the associated Kaplan-Meier curves.

**Table 4.** 90-Day Outcomes of Human Metapneumovirus Relative to Respiratory Syncytial Virus Infection

Outcome	No. with Outcome (matched cohort)		HR (95% CI)^a^	
	hMPV Infection (n = 170)	RSV Infection (Reference) (n = 437)	Unmatched	Matched HR
Hospitalization	31	79	.95 (.64–1.41)	1.00 (.66–1.51)
Death	19	53	.81 (.50–1.31)	.93 (.55–1.56)
Hospitalization and death combined	44	116	.87 (.63–1.21)	.97 (.68–1.37)

Abbreviations: CI, confidence interval; hMPV, human metapneumovirus; HR, hazard ratio; RSV, respiratory syncytial virus.

^a^The HR represents the hazard of the hMPV cohort relative to the RSV cohort.

## DISCUSSION

This propensity-matched cohort study compares outcomes among CLC residents with hMPV and those with influenza virus as well as between those with hMPV and those with RSV. We found similar risks for hospitalization and death with all 3 infections Clinically, NH residents are among those at the highest risk of influenza and RSV infection and the associated complications; now, hMPV shares a similar clinical severity. COPD was notably more prevalent in residents with hMPV relative to influenza or RSV, suggesting a higher burden of lung comorbidity in the hMPV group.

NH residents are at high risk of severe outcomes and death within 90 days from hMPV, influenza, or RSV. Unlike the general older adult population, NH residents inherently meet the risk factors for influenza-related death described by O’Halloran et al [1], including advanced age and comorbid conditions. In addition, the residents’ functional limitations, shared living spaces, and higher prevalence of cognitive impairment further contribute to their heightened vulnerability to infection [20]. The clinical severity of hMPV infection, similar to that of influenza virus infection, necessitates equal vigilance in managing respiratory outbreaks in NHs. The current findings underscore the critical need for increased awareness and proactive management of hMPV in NH populations.

Although hMPV was initially discovered in pediatric populations, it also significantly affects older adults [21]. Past population surveillance studies using multiplex panel analysis have consistently identified hMPV in older populations, indicating its broader impact beyond children. However, only a small number of studies or case series have specifically identified and examined hMPV in NH residents and older populations, finding significant morbidity and 90-day mortality rates associated with the virus in this demographic [3, 4, 22]. Gilca et al [23] studied hMPV in a prospective cohort, and their findings suggested that a cohort of “other respiratory viruses,” including hMPV, RSV, parainfluenza viruses, and coronaviruses, were responsible for respiratory-related disease and deaths within 90 days in NH and elderly populations, particularly in those aged 65–74 or ≥75 years during fall and winter seasons. Jones et al [24] corroborated the significant burden of disease associated with hMPV. In their analysis of a 2018 hMPV outbreak in a West Sydney NH, 10 of 12 elderly persons (aged ≥65 years) with reported hMPV were hospitalized, and 2 died.

Seasonality reports for hMPV come mainly from the younger population, with peaks in late winter and early spring in the United States, the Netherlands, the United Kingdom, Norway, and Finland and late spring and summer in Hong Kong [25]. Occasional summer outbreaks occur, as in a US long-term care facility where 26 residents and 13 staff members were infected [26]. Our systematic analysis of seasonality indicates that hMPV tends to peak later in the year than influenza and RSV, which has important implications for infection control and prevention strategies in NHs [27]. Influenza has long disproportionately affected NH residents and is regularly assessed when symptoms develop; hMPV is likely underdetected but is increasingly included in panel testing. The seasonal nature of hMPV, combined with its potential severe outcomes in vulnerable NH populations, necessitates vigilance during peak seasons. Our study findings also highlight the need for further research to understand the epidemiology and impact of hMPV in this particularly vulnerable population. Future studies should focus on long-term surveillance, effective vaccination strategies, and tailored infection control measures to mitigate the significant health risks posed by hMPV in NH residents.

The current study has many strengths. First, the geographically dispersed CLCs are integrated into a common medical record system, which allows the capture of comprehensive respiratory viral testing across the system. Second, the VA laboratory systems are certified by the Joint Commission and the CLIA which enhance confidence in viral detection. In addition, CLCs have access to infection control professionals, personal protective equipment, and infection control protocols for surveillance and outbreaks, even during the COVID-19 pandemic.

The study also has some limitations, including the predominance of male NH residents, who are generally underrepresented in this population. Although propensity matching was used, it does not account for unobserved variables. Our study is retrospective, with year-to-year variability in the transmission of respiratory viruses among NH residents. The sample size of the hMPV group produced CIs that need further validation in larger studies. Viral testing in NHs was transformed during the study period with the COVID-19 pandemic and commercially available respiratory panel testing, and we were unable to identify specific testing protocols for the population. Furthermore, the COVID-19 pandemic heightened awareness of the importance of testing. As such, hMPV and RSV may be underdetected in our sample. The ECI is dependent on *ICD-10-CM* coding for comorbid conditions and is limited because of possible undercoding or overcoding as well as a lack of a weighting system for comorbid conditions.

In summary, the “underdog” status of hMPV relative to influenza and RSV poses a significant challenge to actively engaging NHs. Our findings related to hospitalization and death add evidence that hMPV should not be considered an “afterthought” once COVID-19, influenza, and RSV have been ruled out. Seasonal analysis suggests that hMPV peaks later in the year than influenza or RSV, indicating the need for targeted infection control measures during these periods. Inclusion of hMPV on respiratory testing panels is crucial to raise awareness. In addition, vaccines for COVID-19, influenza, and RSV have demonstrated benefits for older people, particularly NH residents. Similar investments in hMPV vaccine development may mitigate some of the associated morbidity and 90-day mortality risks. Enhanced infection control measures and continued investigation in comprehensive management approaches, informed by studies like ours, can help reduce morbidity and 90-day mortality rates associated with hMPV, ensuring better health outcomes for NH residents and similar vulnerable populations at risk of hMPV infection. The development and implementation of a vaccine for hMPV could significantly mitigate its impact, especially in vulnerable NH populations.

## Notes

***Financial support.*** This work was supported by Icosavax, a member of the AstraZeneca Group (grant to the Ocean State Research Institute).

***Supplement sponsorship***. This article appears as part of the supplement “Human Metapneumovirus Disease Burden in Older Adults and Implications for Adult Vaccination Strategies,” sponsored by AstraZeneca.
